# Effects of Temperature on the Expression of Two Ovarian Differentiation-Related Genes *foxl2* and *cyp19a1a*

**DOI:** 10.3389/fphys.2018.01208

**Published:** 2018-09-25

**Authors:** Zhi-Gang Shen, Nour Eissa, Hong Yao, Zhi-Gang Xie, Han-Ping Wang

**Affiliations:** ^1^Aquaculture Genetics and Breeding Laboratory, The Ohio State University South Centers, Piketon, OH, United States; ^2^College of Fisheries, Huazhong Agricultural University, Wuhan, China; ^3^Department of Immunology, College of Medicine, University of Manitoba, Winnipeg, MB, Canada; ^4^College of Chemistry and Life Science, Zhejiang Normal University, Jinhua, China

**Keywords:** *foxl2*, *cyp19a*, germ cells, stress, sex differentiation, TSD, GSD

## Abstract

Exposure to stress induces a series of responses and influences a wide range of biological processes including sex differentiation in fish. The present work investigated the molecular and physiological response to thermal stress throughout the early development stage covering the whole period of sex differentiation of bluegill, *Lepomis macrochirus*. Larvae were treated using three temperatures, 17, 24, and 32°C from 6 to 90 days posthatching (dph) in 30-L round tanks. There is no significant difference of the sex ratio and survival among the three temperature groups in the geographic population used in this study. Two ovarian differentiation-related genes *foxl2* and *cyp19a1a* were detected at 7 dph suggesting that these genes have already played a role prior to sex differentiation. The expression of *foxl2* reached the peak and was thermosensitive just prior to the onset of ovarian differentiation at 27 dph. Histological examination displayed that the proliferation of germ cells and ovarian differentiation were delayed at the low-temperature treatment (17°C) at 97 dph compared with higher temperatures. In conclusion, the water temperature regulates the sex differentiation of bluegill through modulation of the expression of *foxl2* and *cyp19a1a.* A comparative study of the expression profile of sex differentiation-related genes in species will shed light on the evolution of sex-determination mechanisms and the impact of stress on sex differentiation.

## Introduction

Sex could be initiated by genetic or environmental signals (e.g., temperature), and the sex-determining gene(s) vary among fishes (Kikuchi and Hamaguchi, 2013; Bachtrog et al., 2014; Shen and Wang, 2014, 2018a; Mei and Gui, 2015; Shen et al., 2016; Wang and Shen, 2018). The sex differentiation-related genes are relatively conserved (Siegfried, 2010; Kobayashi et al., 2013; Shen and Wang, 2014). Several studies explored the molecular aspects of sex differentiation, such as transcription factor genes (*foxl2*, *dmrt1*, and *sox9*) and steroidogenic-related genes (*cyp19a1*, *amh*, and *sf-1*). This line of research is important because comparative approaches across diverse taxa expand the understanding of the molecular function and interactions, cellular behaviors (e.g., germ cells), and evolution of signaling pathways in the realization of phenotypic sex.

**FIGURE 1:**
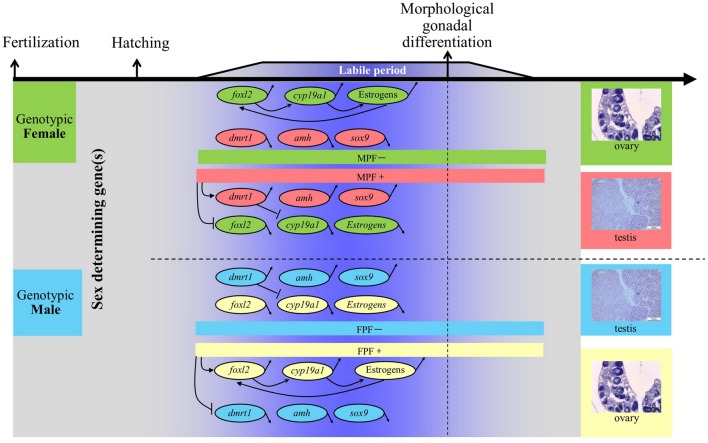
General schematic diagram of molecular players involved in sex differentiation of gonochoristic teleost with genetic sex determination. Note that this represents a “typical” sex-differentiation pathway, while large-scale variations exist with respect to timing and sexual dimorphism of expression, as well as regulatory mechanisms. MPF, male producing/promoting factors, including androgens, estrogen receptor antagonists, aromatase inhibitors, male producing temperature, etc. FPF, female producing/promoting factors, including estrogens, androgen receptor antagonists, female producing temperature, etc. Upward/downward arrows indicate the expression of specific gene is upregulated/downregulated, respectively. Arrows pointing to specific gene indicate that the expression is upregulated. Inhibiting symbols pointing to specific gene indicate that the expression is downregulated. Data refer to D’Cotta et al. (2007); Vizziano et al. (2007); Wang et al. (2007, 2010); Yamaguchi et al. (2007); Baron et al. (2008); Ijiri et al. (2008); Liu et al. (2010); Siegfried (2010); Poonlaphdecha et al. (2013); and Shen and Wang (2014).

A general schematic diagram represents the molecular players that are involved in sex differentiation of teleosts, which employ genetic sex determination (GSD) (**Figure 1**). Most of the interactions between ovarian differentiation-related genes (e.g., *foxl2* and *cyp19a1*) and testicular differentiation-related genes (e.g., *dmrt1*, *amh*, and *sox9*) are elusive. However, the expression patterns are somewhat clear. Of these candidate genes, *foxl2* and *cyp19a1a*, have received considerably more attention. The *foxl2* is a forkhead domain transcription factor, which is required for granulosa cell differentiation and ovarian maintenance (Schmidt et al., 2004; Baron et al., 2005; Uhlenhaut and Treier, 2006; Corpuz et al., 2010; Kashimada et al., 2011; Georges et al., 2013). Sexual dimorphic expression of *foxl2* during sex differentiation has been found in all species investigated except American Alligator *Alligator mississippiensis*, including species with either temperature-dependent sex determination (TSD) or GSD (**Table 1**). The expression of foxl2 also generally displays a parabola trend with a climax at the critical point of or right before sex differentiation. Regarding TSD, its expression displays a thermo-sensitive pattern, with female-producing (promoting) temperature increasing and male-producing temperature decreasing its expression (**Table 1**). The pivotal role of the *foxl2* gene in fish ovarian differentiation has been confirmed by the evidence that *foxl2* could upregulate aromatase gene transcription directly by binding to the promoter region of *cyp19a1a*, or indirectly through interacting with *sf-1* (steroidgenic factor 1, also known as *Ad4BP* or *Nr5a1*) (Wang et al., 2007; Yamaguchi et al., 2007). Furthermore, *foxl2* is regulated by water temperature and involved in temperature-induced sex reversal in Japanese flounder with TSD (Yamaguchi et al., 2007). Recently, a research study showed that XX female medaka with disrupted *foxl3* (a paralog of *foxl2*) developed functional sperm (Nishimura et al., 2015), suggesting the crucial role of *foxl3* in female fate.

**Table 1:** *foxl2* expression profile.

Species	Express before MGD	Sexual dimorphic expression during SDi	Reverse parabola expression	Thermo-sensitive	Population used	Positively correlated with *cyp19a*	Regulate *cyp19a* directly	Regulate *cyp19a* by *sf-1*	SDe mode	Reference
Nile tilapia	√	√	×	NS	XX female	√	NS	NS	GSD+TE	(Ijiri et al., 2008)
					XY male					
	√	√	NS	NS	XX female	√	√	√		(Wang et al., 2007)
					XY male					
					XY female					
					XX male					
Japanese flounder	√	√	NS	√	XX female	√	√	NS	GSD+TE£	(Yamaguchi et al., 2007)
Medaka	×	√	NS	NS	Mixed sexes^#^	NS	NS	NS	GSD+TE	(Nakamoto et al., 2006)
Airbreathing catfish	√	√	√	NS	Mixed sexes	√	NS	NS	GSD+TE	(Sridevi and Senthilkumaran, 2011)
Rainbow trout	√	√	NS	NS	XX female	√	NS	NS	GSD+TE	(Baron et al., 2004)
					XY male					
	√	√	√	NS	XX female	×	NS	NS		(Vizziano et al., 2007)
					XY male					
Willow minnow	√	NS	NS	NS	Mixed sexes	NS	NS	NS	GSD+TE£	(Ashida et al., 2013)
Zebrafish	√	√	√	NS	Mixed sexes§	NS	NS	NS	GSD+TE	(Hossain, 2010)
*Oryzias luzonensis*	√	√	NS	NS	Mixed sexes§	NS	NS	NS	GSD+TE	(Nakamoto et al., 2009)
Pacific oyster	NS	NS	√	√	Mixed sexes	NS	NS	NS	GSD+TE	(Santerre et al., 2013)
American alligators	√	×	NS	×	Mixed sexes^∫^	NS	NS	NS	TSD	(Janes et al., 2013)
Snapping turtle	√	√	√	√	Mixed sexes^∫^	NS	NS	NS	TSD	(Shoemaker-Daly et al., 2010)
Red-eared slider turtle	√	√	NS	√	Mixed sexes^∫^	NS	NS	NS	TSD	(Loffler et al., 2003)
	√	√	NS	√	Mixed sexes^∫^	NS	NS	NS		(Shoemaker et al., 2007)
Bluegill sunfish	√	NS	√	√	Mixed sexes	NS	NS	NS	GSD or TSD or GSD+TE	Present work


*MGD, morphological gonad differentiation; SDi, sex differentiation; SDe, sex determination; GSD, genetic sex determination; TSD, temperature-dependent sex determination; TE, temperature effects. √, yes; ×, no; NS, not studied. ^#^, genetic sex could be identified by PCR-based strategy. ^§^, sex could be distinguished by a molecular marker. ^∫^, mono-sex was produced at female/male producing temperature. ^£^, Sex determination has been claimed as GSD+TE or TSD, here we refer to Ospina-Álvarez and Piferrer (2008).*

The *cyp19a1a* gene is expressed in the undifferentiated, differentiating, and differentiated gonads, as well as organs of adult fish and encodes aromatase, which is a key enzyme during the formation of estrogens from androgens (Piferrer and Blázquez, 2005; Guiguen et al., 2010; Shen and Wang, 2014). The expression of *cyp19a1a* is increased prior to morphological sex differentiation in the gonochoristic fish species and is tightly related with temperature-induced feminization (Siegfried, 2010; Shen and Wang, 2014). The *cyp19a1a* is also involved in natural sex reversal of hermaphroditic species (Huang et al., 2009).

Changes in the water temperature result in various physiological and molecular stress responses to maintain the essential biological functions in fish and other aquatic species (Eissa and Wang, 2013, 2016; Eissa et al., 2017, 2018). Bluegill sunfish (*Lepomis macrochirus*) belongs to Centrarchidae (Near and Koppelman, 2009) and receives much attention because of the extraordinary biological characteristics, such as sex-determining mechanism (Wang et al., 2014; Shen et al., 2016), hybridization (Shen and Wang, 2018b), and alternative mating tactics (Gross and Charnov, 1980; Dominey, 1981; Garner and Neff, 2013). These biological features influence population sex ratio solely or interactively and make bluegill to be an excellent organism for investigation of interactions, population dynamics, speciation, and sexual selection.

To our knowledge, molecular pathways involved in sex differentiation of bluegill have not been studied yet. Therefore, the present study aimed to investigate the molecular players that can regulate the sex differentiation of bluegill.

## Materials and Methods

### Fish, Experimental Design, and Sampling Points

The experiments were conducted in accordance with the ethical standards and according to the national and international guidelines. This study and all experimental procedures involving animals were performed according to the protocol that was approved by The Ohio State University Institutional Animal Care and Use Committee.

Larvae were produced according to the procedures of bluegill out-of-season spawning established in the Aquaculture Genetics and Breeding Lab at The Ohio State University South Centers (Gao et al., 2009; Wang et al., 2014). Newly hatched larvae were reared at 24 ± 1°C in 400-L round tanks (spawning tank). Five-days posthatching (dph) larvae were transferred to 30-L round tanks with aeration and flow-through water. The experiment consisted of three different temperature treatments: 17, 24, and 32°C. On the next day of the transfer on 6 dph, water temperatures were adjusted to targeted ones within a week period, using cold well water (17°C), heated water (24°C), or heated water plus heaters (32°C). Temperature treatments were carried out in triplicates, each having 500 larvae. After 90 dph, water temperatures for all groups were gradually adjusted to 24°C and then maintained at 24 ± 1°C until the end of the experiment. Photoperiod was adjusted at 16 h light and 8 h dark during the experiment.

Fish were periodically sacrificed with an overdose of MS-222 (300 mg kg^-1^) and whole fish samples were collected at 7, 17, 27, 37, and 57 dph from each treatment replicate. These sampling points cover the critical period of sex differentiation in bluegill according to Gao et al. (2009). The whole fish samples were stored in RNA*later*^®^ Stabilization Solution (Ambion^®^, Life Technologies, United States) at 4°C overnight and transferred to -80°C until further analysis. Moreover, six fish from each temperature group were overanesthetized and fixed in 10% formalin (Anatech Ltd., MI, United States) at 57 and 97 dph for histological examination.

### RNA Extraction and cDNA Synthesis

Total RNA was extracted from the individual whole fish samples (except 7 dph, for which 30 samples taken from each treatment replicate were pooled) by homogenization in TRIzol^®^ Reagent (Ambion^®^, Life Technologies, United States) following the manufacturer’s procedure. Concentrations were assessed by spectrophotometry (NanoDrop 1000, Thermo Fisher Scientific Inc., United States). The quality of the RNA was checked by electrophoresis on 1% agarose gel (SYBR^®^ Green stain) and by A_260 nm_/A_280 nm_ ratios. Isolated RNA samples were then treated with RQ1 RNase-Free DNase (Promega Corporation) according to the manufacturer’s procedure. Total RNA (1 μg) was reverse-transcribed to cDNA with high-capacity cDNA Reverse Transcription Kits (Applied Biosystems^®^, Life Technologies, United States) following the manufacturer’s instructions.

### Quantitative and Qualitative Gene Expression Analysis

Real-time reverse transcription polymerase chain reaction (RT-PCR) reactions were performed with an ABI 7500 real-time PCR System (7500 Software v2.0.6, Applied Biosystems^®^) using the SYBR^®^ Select Master Mix (Applied Biosystems). Primer sequences (5′–3′) used in the present study were: *foxl2* forward CAGAGCATGGCGCTCCCCAGC, reverse AACGCCGAGTGTTTGGTCTCGTG (target length 227 bp); *18S rRNA* forward AGGAATTGACGGAAGGGCAC, reverse GGTGAGGTTTCCCGTGTTGA (target length 73 bp); and *cyp19a1a* forward ACTCACTTAGACGGCTTGGACAG, reverse CACTCACAGGTACACCCAGGAAG (target length 109 bp). Primers were designed according to available sequences. The PCR conditions were optimized through gradient testing for the best annealing temperature, combining melt curve and electrophoresis for best primer concentration, etc. before quantitative analysis. Data analysis using the 2^-ΔΔ*C*_t_^ method was applied in the study (Livak and Schmittgen, 2001; Schmittgen and Livak, 2008). Therefore, primer PCR efficiencies (E) were evaluated with the standard curve method, and only primers for *foxl2* (*E* = 104.048%) and *18S rRNA* (*E* = 106.331%) were adopted for qRT-PCR. Primers for *cyp19a1a* were only used for qualitative analysis (express or not). The samples from 7 dph larvae were applied as a calibrator. Hence, exponential power for the fold change of gene expression deduced from 2^-ΔΔ*C*_t_^ formula can be expressed as follows:

$$-\Delta \Delta Ct=-\left[(C_{t,foxl2}-C_{\text{t, 18S rRNA}})Time\,\;X-(C_{t,foxl2}-C_{\text{t, 18S rRNA}})Time\,\;0\right]$$

Where C_t, *foxl*2_ and C_t,18SrRNA_ denote the threshold cycles (C_t_) for the target gene *foxl2* and reference gene *18S rRNA*, respectively; Time *X* denotes different sampling point; Time 0 denotes the calibrator sampling point, which was 7 dph in the study.

Triplicates were run in a MicroAmp^®^ Optical 96-Well Reaction Plate (Applied Biosystems^®^) in a final volume of 20 μL, which consisted of 10 μL SYBR^®^ Select Master Mix (2×), 300 nM of each primer (final concentration), 1 μL cDNA template (from 50 ng RNA), and double distilled water. Cycling parameters were 50°C for 2 min, 95°C for 2 min, followed by 40 cycles of amplification at 95°C for 15 s, annealing at 60°C for 15 s, and extension at 72°C for 1 min. Finally, the temperature melt curve step was performed at the end of the amplification phase to check non-specific amplification. In addition, electrophoresis of PCR products was performed to check early expression of *cyp19a1a*, *foxl2*, and *18S rRNA* qualitatively, non-specific amplification, and the length of target genes. Raw data were exported from 7500 Software v2.0.6 in Excel format for further statistical analysis.

### Histological Analysis and Sex Identification

Histological sectioning was carried out to investigate the effects of temperature on sex differentiation. The fish heads were removed prior to the branchiostegal membrane, and the tails were removed after the anal opening. The remaining middle portion samples were transferred into 70% ethanol, dehydrated, and embedded in paraffin. Cross- or longitudinal sections of 6–7 μm were cut, and slices were stained with H&E, and counterstained with eosin. Tissue slices were examined and photographed under a light microscope with an imaging system (Olympus MicroSuite FIVE, FL, United States). Development stages and cellular identification were based on descriptions and photographs from Gao et al. (2009). All fish were overanesthetized and subjected to sex identification under microscope using the gonadal squash method at the age of 170–190 dph.

### Statistical Analysis

Differences in mean gene expression among three different temperature treatments and at different days posthatching were analyzed by two-way analysis of variance (Two-Way ANOVA, General Linear Model). Before the analysis, data were tested for normality (Shapiro-Wilk test) and gene expression levels ln (natural log) transformed to ensure the homogeneity of variances. Differences of sex ratio among the three temperature groups or deviation from the balanced sex ratio of 1:1 were analyzed by Chi-squared test. Statistical analyses were performed with IBM SPSS Statistics Version 19. Differences were considered statistically significant when *P* < 0.05.

## Results

### Early Expression of *foxl2* and *cyp19a1a*

**FIGURE 2:**
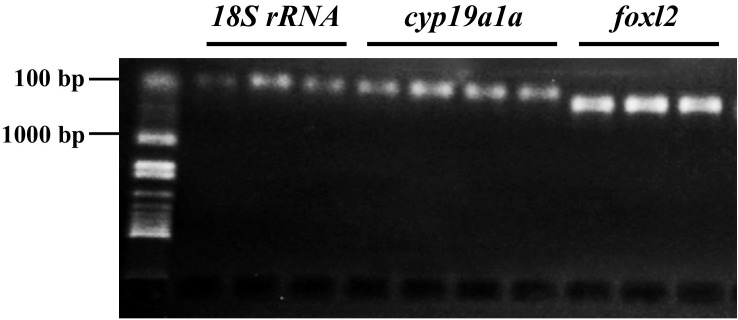
Expression of sex differentiation-related gene *foxl2* and *cyp19a1a* at 7 and 17 days posthatching (dph) in bluegill *L. macrochirus*. The leftmost sample in sample group of each gene was from 7 dph individual, and the rest two or three were from 17 dph individuals. The leftmost column was molecular size marker.

Both genes related to sex differentiation, *foxl2* and *cyp19a1a*, were expressed as early as 7 dph (at 5.5 mm total length, **Figure 2**).

### Temporal Expression of *foxl2* and Temperature Effects

The RT-qPCR standard curves exhibited a significant linear relationship between the values of threshold cycle (CT) and the gene copy number in both *foxl2* and *18S rRNA* genes (**Supplementary Figure S1**). The PCR efficiencies of both genes were high (104.048 and 106.331%), indicating the reliability of primers for quantification of these genes.

**FIGURE 3:**
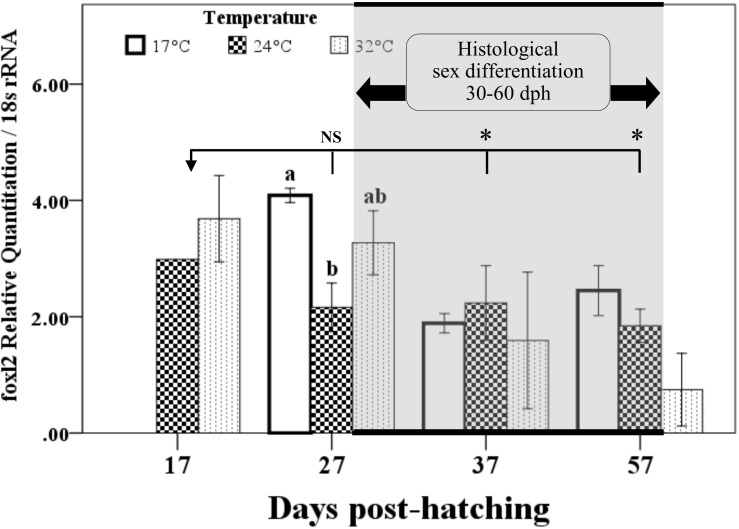
Temporal expression of *foxl2* and temperature effects in the early life stage of bluegill *L. macrochirus* normalized against *18S rRNA* measured by real-time RT-PCR. Results are mean ± SE. Different letters in 27 days posthatching (dph) sampling point indicate significant difference among temperature treatments. Asterisks denote significant difference between 17 and 37 dph groups or 17 and 57 dph groups; NS, no significance between 17 and 27 dph group when pooling different temperature treatments together. Gray shadow area indicates that histological sex differentiation occurs between 30 to 60 dph according to our previous study (Gao et al., 2009).

Expression of *foxl2* increased dramatically from 7 to 17 dph, stabilized from 17 to 27 dph, and then started to decrease significantly at 37 dph (**Figure 3**). Remarkable effects of temperature on *foxl2* expression were observed at 27 dph, where *foxl2* expression was the highest in 17°C treatment and the lowest in 24°C treatment. No dph–temperature interaction on *foxl2* expression was observed. Furthermore, the expression of *foxl2* of four individuals at 37 dph in 32°C treatment was dramatically lower than other groups, even significantly lower than the samples at 7 dph.

### Gonadal Histology

**FIGURE 4:**
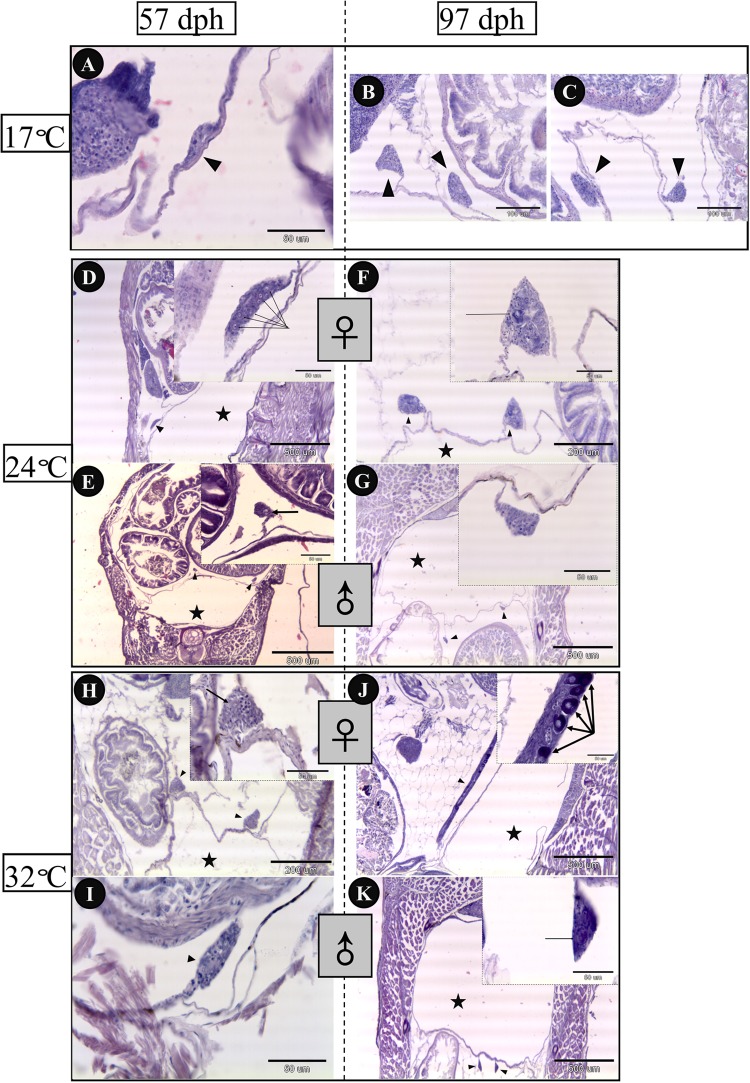
Gonadal histology of bluegill *L. macrochirus* in three temperature-treatment groups (17, 24, and 32°C) at 57 and 97 days posthatching (dph). Solid triangles indicate gonads. Five-pointed stars denote the place of swim bladder. 17°C group: **(A)** undistinguishable gonad at 57 dph. **(B,C)** undistinguishable gonad at 97 dph. 24°C group: **(D)** presumptive ovary at 57 dph, arrows indicate several germ cells. **(E)** presumptive testes at 57 dph, arrow indicates germ cell. **(F)** ovary at 97 dph, arrow points to peri-nucleolus oocyte. **(G)** testes at 97 dph. 32°C group: **(H)** presumptive ovaries at 57 dph, arrow indicates germ cell. **(I)** presumptive testis at 57 dph. **(J)** ovary at 97 dph, arrows point to peri-nucleolus oocytes. **(K)** testes at 97 dph, arrow indicates germ cell.

Gonadal histology of bluegill larvae at different rearing temperatures was investigated from 57 to 97 dph in the present work (**Figure 4**). Sex could be distinguished in the 24°C (**Figures 4D,E**) and 32°C treatment groups (**Figures 4H,I**), but not in the 17°C group (**Figure 4A**) at 57 dph. Ovaries differentiated earlier, in which primordial germ cells overnumbered on 57 dph in comparison with presumptive testis of larvae reared at 24 and 32°C. In addition, ovaries developed dramatically from 57 to 97 dph in light of which the perinucleolus stage oocytes had been observed at 97 dph both in the 24 and 32°C groups (**Figures 4F,J**). In contrast, testes did not display obvious changes from 57 to 97 dph (**Figures 4G,K**) in any treatment group, other than size increase. Ovarian development was inhibited by low temperature because sex could not be identified up to 97 dph in the 17°C group (**Figures 4B,C**).

## Discussion

### The *foxl2* and *cyp19a1a* Play a Role Before the Onset of Morphological Ovarian Differentiation

In this study, we showed that the expression of the two sex differentiation-related genes, *foxl2* and *cyp19a1a*, were detected in bluegill larvae at 7 dph (**Figure 2**), which is much earlier than morphological gonadal differentiation (Gao et al., 2009). The *foxl2* gene is a regulator of the aromatase gene (Pannetier et al., 2006; Wang et al., 2007). In tilapia, alteration of the expression of *foxl2* could cause sex reversal of XX females to males, while overexpression of *foxl2* leads to degeneration of the testicular structure and a stimulation of estrogen production (Wang et al., 2007). Estrogen or aromatase inhibitor treatment during the labile period of sex differentiation suggested that *foxl2* is regulated through a positive feedback mechanism (Baron et al., 2004; Liu et al., 2007): *foxl2*–aromatase gene–aromatase–estrogen–*foxl2* (**Figure 1**). The expression of *foxl2* was detected before morphological gonadal differentiation in all investigated species except medaka, indicating its role prior to sex differentiation. Furthermore, expression of *foxl2* is significantly increased just at the onset of or during sex differentiation, strongly supporting the notion that *foxl2* plays an essential role in ovarian differentiation.

The *cyp19a1a* and estrogens are key players during the ovarian differentiation in fish (Piferrer and Blázquez, 2005; Guiguen et al., 2010; Shen and Wang, 2014). Expression of *cyp19a1a* has been detected prior to sex differentiation (Shen and Wang, 2014). The present work showed that *cyp19a1a* is expressed prior to sex differentiation, suggesting its essential role in ovarian differentiation. However, future studies are warranted to focus on the underlying mechanism connecting environmental factors (e.g., water temperature) and *cyp19a1a* expression.

### Expression of *foxl2* Was Thermo-Sensitive

The expression of *foxl2* was thermo-sensitive and significant differences were found between temperature treatments at 27 dph prior to the onset of morphological sex differentiation (**Figure 3**). However, no significant differences have been detected in sex ratio among the three temperature treatments. The present study shows that the low temperature treatment (17°C) suppressed the growth and inhibited gonad development as well (**Figures 4A–C**). The progress of sex differentiation is more dependent on length than on age (Piferrer et al., 2005; Gao et al., 2009). Furthermore, the growth is related to phenotypic sex in European sea bass (Saillant et al., 2001) and assigned as growth-dependent sex differentiation (Ospina-Álvarez and Piferrer, 2008). The so-called growth-dependent sex differentiation was observed in southern brook lamprey (*Ichthyomyzon gagei*), American eel (*Anguilla rostrata*), and olive flounder (Kraak and De Looze, 1992; Ospina-Álvarez and Piferrer, 2008). This study suggests that bluegill from different geographic locations might have different genotypes and sensitivity to environmental temperatures. Therefore, sex differentiation of bluegill could be more complicated and mysterious because of growth-dependent sex differentiation or TSD.

Thermosensitivity of *foxl2* has been reported in Japanese flounder (*Paralichthys olivaceus*) in which temperature treatment (18 or 27°C) could result in 100% sex reversal. The expression of *foxl2* also varied with rearing temperatures in Pacific oyster (Santerre et al., 2013). Therefore, *foxl2* is the key regulator for the sex differentiation. In species with TSD, no consistent genetic differences exist between female and male (Valenzuela et al., 2003; Conover, 2004; Ospina-Álvarez and Piferrer, 2008; Shen and Wang, 2014). Moreover, in TSD species, the ambient temperatures trigger the gonadal differentiation (Shoemaker and Crews, 2009). In species with GSD (or with GSD+TE), the initiation of sex differentiation is activated by sex-determining gene(s). Therefore, the comparative analysis of the expression profile of *foxl2* and other sex differentiation-related genes will further enhance the understanding of the evolution of sex-determination mechanisms.

### Thermal Stress on *foxl2* Expression and Ovarian Development

Temperature has an extensive influence on an organism, including a series of physiological and biochemical processes, which results in a specific phenotype (Mccue, 2004). The water temperature influences developmental, behavioral, physiological, and morphological traits in many kinds of animals, especially in reptiles and fish (Mccue, 2004; Rhen and Lang, 2004; Booth, 2006). However, there is poor understanding of how temperature transduces molecular signals into the body and affects sex differentiation-related genes (Shen and Wang, 2014, 2018a). The present study exhibits that low temperature increased the expression of *foxl2* at 27 dph, while low-temperature treatment suppressed ovarian development as revealed by histological examination (**Figure 3**). In Pacific oyster, the best growth performance was observed between 27 and 32°C (Rico-Villa et al., 2009). However, low temperature (18°C) suppressed gonadal differentiation and increased expression of *foxl2*, while high temperature promoted gonadal differentiation and decreased expression of *foxl2* (Santerre et al., 2013). Bluegill has better growth performance at water temperature of 28 and 32°C (Beitinger and Magnuson, 1979). The inconsistency in the current findings can be explained by that *foxl2* is only thermo-sensitive during the critical period of sex differentiation (around 27 dph). Moreover, *foxl2* is not involved in TSD in bluegill because its elevated expression did not affect sex ratio in the low-temperature group. Therefore, the expression changes of *foxl2* might be a result of physiological development. Temperature effects on *foxl2* expression during critical period of sex differentiation or its effects on germ cells have been investigated (Lee et al., 2009; Pandit et al., 2015). However, the effects of *foxl2* on both of them have not been reported yet. Further studies are required to address the expression difference of sex differentiation-related genes, e.g., *foxl2*, *cyp19a1a*, *dmrt1*, and *amh*, in species with both GSD and TSD and the roles of these genes in the transition of sex-determining mechanisms.

### Thermal Stress on Primordial Germ Cells and Sex Differentiation

Mitotic proliferation of primordial germ cells and formation of the ovarian cavity are acceptable indictors of the initialization of ovarian differentiation in teleost (Nakamura et al., 1998). This study shows that the germ cells of bluegill in putative ovaries outnumbered those in putative testes (**Figures 4D,E,H,I**). This study shows delayed proliferation and reduced numbers of germ cells in the low-temperature treatment (17°C) and morphological sex differentiation had not been detected up to 97 dph (**Figure 4**). In pufferfish (*Takifugu rubripes*), high-temperature treatment during early gonadal development induced germ cell degeneration and masculinization of ovarian somatic cells (Lee et al., 2009). In Nile tilapia, high temperature treatment (37°C) also resulted in a permanent depletion of germ cells indicating the involvement of germ cells in temperature-induced sex reversal (Pandit et al., 2015). Furthermore, germ cell ablations have confirmed the involvement of germ cells in sex differentiation in zebrafish (*Danio rerio*) and medaka (Slanchev et al., 2005; Kurokawa et al., 2007; Siegfried and Nüsslein-Volhard, 2008) and no involvement in loach (*Misgurnus anguillicaudatus*) and goldfish (*Carassius auratus*) (Fujimoto et al., 2010; Goto et al., 2012). Germ-cell-deficient genotypic female in zebrafish developed as sterile males, which are able to mate with normal females and induce them to lay eggs (Slanchev et al., 2005). These findings may be of practical importance for fish population control or fisheries management through production of genetically modified male (even YY super-male) and/or disturbing the germ cells, which may cause rapid decrease in population.

## Conclusion

The present work investigated the molecular and physiological response to thermal stress throughout the early development stage of sex differentiation in bluegill. Our results suggest that the two ovarian differentiation-related genes, *foxl2* and *cyp19a1a*, play a role prior to sex differentiation of bluegill. The *foxl2* may be involved in TSD in some species, but not in others. Further studies will focus on the comparative analysis of molecular network of sex differentiation.

## Author Contributions

Z-GS and H-PW conceived and designed the experiments. Z-GS, NE, HY, and Z-GX performed the experiments. Z-GS analyzed the data and wrote the paper. H-PW and HY contributed reagents, materials, and analysis tools. H-PW, NE, and Z-GX revised the draft version. All authors have read and approved the manuscript.

## Conflict of Interest Statement

The authors declare that the research was conducted in the absence of any commercial or financial relationships that could be construed as a potential conflict of interest.

## Supplementary Material

FIGURE S1 Standard curves of *foxl2* and *18S rRNA* gene indicating a linear relationship between threshold cycle (CT) and gene copy number (concentration, copies μL^-1^). Eff %, PCR efficiency.
