# Supplementary figures and images for: Effects of Temperature on the Expression of Two Ovarian Differentiation-Related Genes foxl2 and cyp19a1a
Source: Front Physiol. 2018 Sep 25;9:1208. doi: 10.3389/fphys.2018.01208 (PMC6190877; doi:10.3389/fphys.2018.01208)

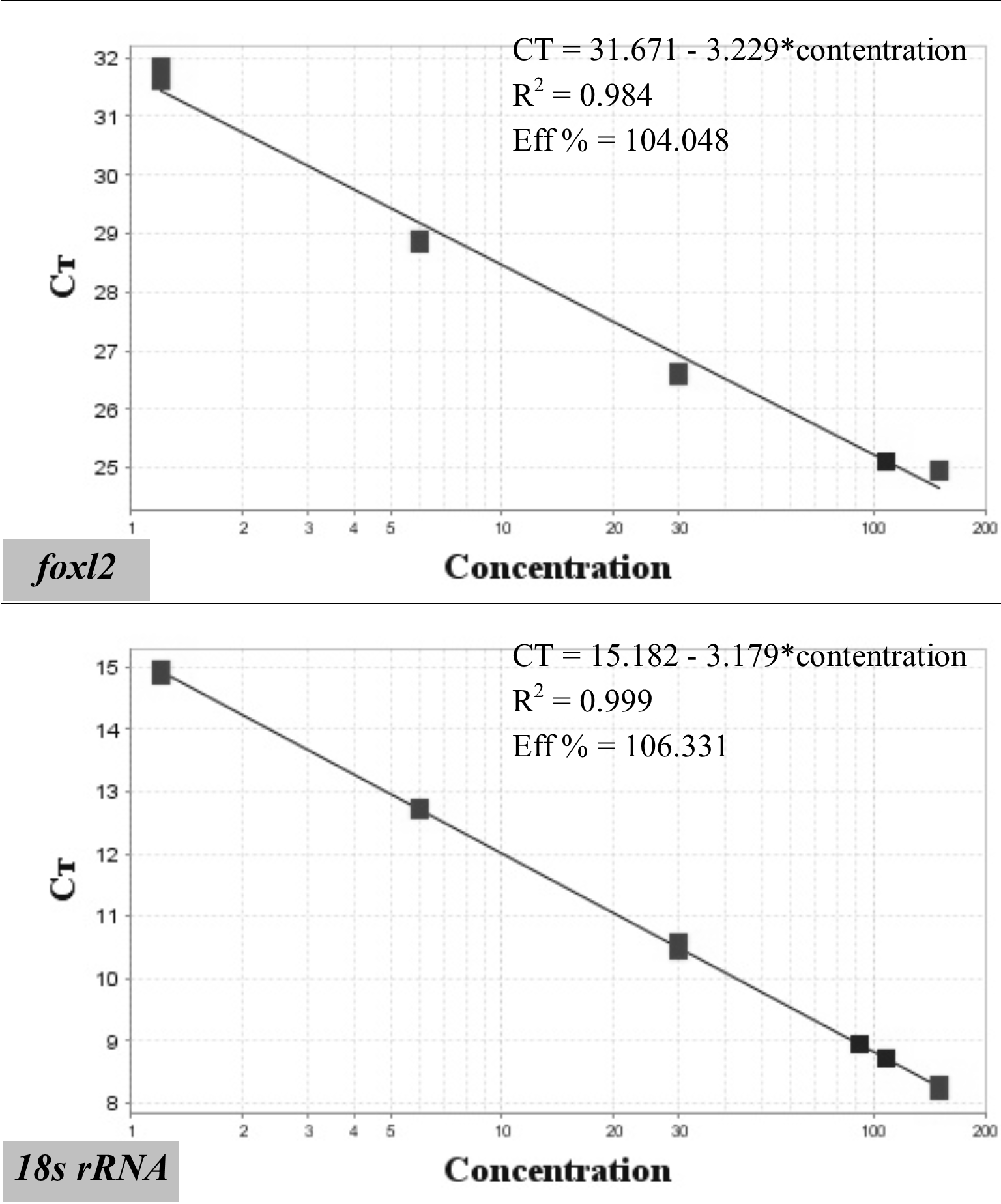

Supplement: FIGURE S1 — Standard curves of foxl2 and 18S rRNA gene indicating a linear relationship between threshold cycle (CT) and gene copy number (concentration, copies μL-1). Eff %, PCR efficiency. [file Image_1.TIF]
